# Evaluating the performance of a low-cost mobile phone attachable microscope in cervical cytology

**DOI:** 10.1186/s12905-020-00902-0

**Published:** 2020-03-25

**Authors:** Ali Naqvi, Niti Manglik, Ellen Dudrey, Cynthia Perry, Zuber D. Mulla, Jorge L. Cervantes

**Affiliations:** 1 Department of Medical Education, Paul L. Foster School of Medicine, Texas Tech University Health Sciences Center El Paso, 5001 El Paso Dr, El Paso, TX 79905 USA; 2 Department of Pathology, Texas Tech University Health Sciences Center, El Paso, TX USA; 3 Department of Obstetrics and Gynecology and Office of Faculty Development, Paul L. Foster School of Medicine, Texas Tech University Health Sciences Center El Paso, El Paso, TX USA

**Keywords:** Foldscope, Cervical cytology, Smart phone, Agreement, Accuracy

## Abstract

**Background:**

Cervical cancer remains a global health problem especially in remote areas of developing countries which have limited resources for cervical cancer screening. In this study, we evaluated the performance of a low-cost, smartphone attachable paper-based microscope when used for classifying images of cervical cytology.

**Methods:**

Cervical cytology samples included: 10 Normal, 10 Low-grade squamous intraepithelial lesion (LSIL), 10 High-grade squamous intraepithelial lesion (HSIL), and 10 Malignant Pap Smears. The agreement between conventional microscopy vs. Foldscope imaging was calculated using a weighted kappa coefficient. A confusion matrix was created with three classes: Normal, LSIL, and HSIL/malignant, to evaluate the performance of the Foldscope by calculating the accuracy, sensitivity, and specificity.

**Results:**

We observed a kappa statistic of 0.68 for the agreement. This translates into a substantial agreement between the cytological classifications by the Foldscope vs. conventional microscopy. The accuracy of the Foldscope was 80%, with a sensitivity and specificity of 85 and 90% for the HSIL/Mal category, 80 and 83.3%, for LSIL, and 70 and 96.7% for Normal.

**Conclusions:**

This study highlights the usefulness of the Foldscope in cervical cytology, demonstrating it has substantial agreement with conventional microscopy. Its use could improve cytologic interpretations in underserved areas and, thus, improve the quality of cervical cancer screening. Improvements in existing limitations of the device, such as ability to focus, could potentially increase its accuracy.

## Background

High resolution cell phone cameras and microscope lens technology have become widely available and affordable over the recent decade [1, 2]. Despite the significant reduction in the cost of microscope imaging in recent years, many underdeveloped regions of the world still lack the finances to utilize such advances. Smart phones have seen notable technological adaptation, with attachable microscope lenses for smart phone cameras, increasing the magnification of the acquired image, that may rival conventional laboratory diagnostic microscopes at a fraction of the cost [3]. In addition, image editing and processing applications pre-installed on phones allow for immediate manipulation and transmission of images. These advancements may offer a more cost effective solution to the problems of microscopic imaging in the developing world.

Cervical cancer remains a global health problem [4] especially in remote areas of developing countries [5, 6]**.** Current cervical cancer screening is based on the pap smear cytology and has led to significant reduction in the incidence and death related to cervical cancer [6, 7]. In depth understanding of the natural history of human papillomavirus (HPV) infection and related cervical neoplasia has led to the search of biomarkers which can improve the cervical cancer screening process. Some promising biomarkers can detect cervical dysplasia with higher potential to progress to invasive carcinoma and help supplement the Pap smear results, especially in non-diagnostic and inconclusive cases. These biomarkers, however, are mainly used on cervical biopsy specimens and the results might not be easy to interpret in cytology specimens with dispersed cell population [8]. In developed countries pap smears are also supplemented by HPV DNA testing in certain clinical settings. This test, however, not be available or affordable in places with limited resources.

Affordable and effective microscope imaging technology has the potential to significantly impact disease detection in places where diagnostic laboratories are scarce. Efforts to utilize phone attachable microscopes in diagnostic microscopy are already underway, but reports have been limited to parasitic identification [9] with just a single published report examining this technology’s potential in dealing with cervical cytology [10]. In remote areas, particularly in the developing world, the Foldscope could offer a low cost method for the diagnosis of cervical cytology.

In this study we evaluated the performance of a low-cost, smartphone attachable paper-based microscope when used for classifying images of cervical cytology. Besides evaluating how classification of cytological changes using this device compares to conventional microscopy, we also calculated epidemiological parameters of sensitivity and specificity. Our ultimate goal is to test whether images from the Foldscope can be used by pathologists to classify cervical cytology.

## Methods

The study protocol was reviewed by the Texas Tech University Health Sciences Center El Paso (TTUHSC El Paso) Institutional Review Board (IRB) for the Protection of Human Subjects and was deemed exempt from formal IRB review (# E19046).

### Cervical cytology slides

De-identified Thinprep Pap smears were obtained from the archives of the University Medical Center of El Paso Department of Pathology. A total of 40 representative slides of cervical cytology were selected. The samples included: 10 Normal (i.e. negative for intraepithelial lesions of malignancy), 10 Low-grade squamous intraepithelial lesion (LSIL), 10 High-grade squamous intraepithelial lesion (HSIL), and 10 Malignant (squamous cell carcinoma, adenocarcinoma, and other malignant neoplasm). All Pap smears were screened by cytotechnologists and pathologists using the Bethesda Classification and the reported diagnosis was accepted as the ‘gold standard’ for our study [11]. These slides were retrieved from the archives, and no staining was performed in this study. Samples were randomly divided into two sets of 20 (Group 1 and Group 2) to be reviewed by two pathologists for imaging and classification. The pathologists were blinded to the specific breakdown of representative slides.

### Image acquisition and classification

**Fig. 1:**
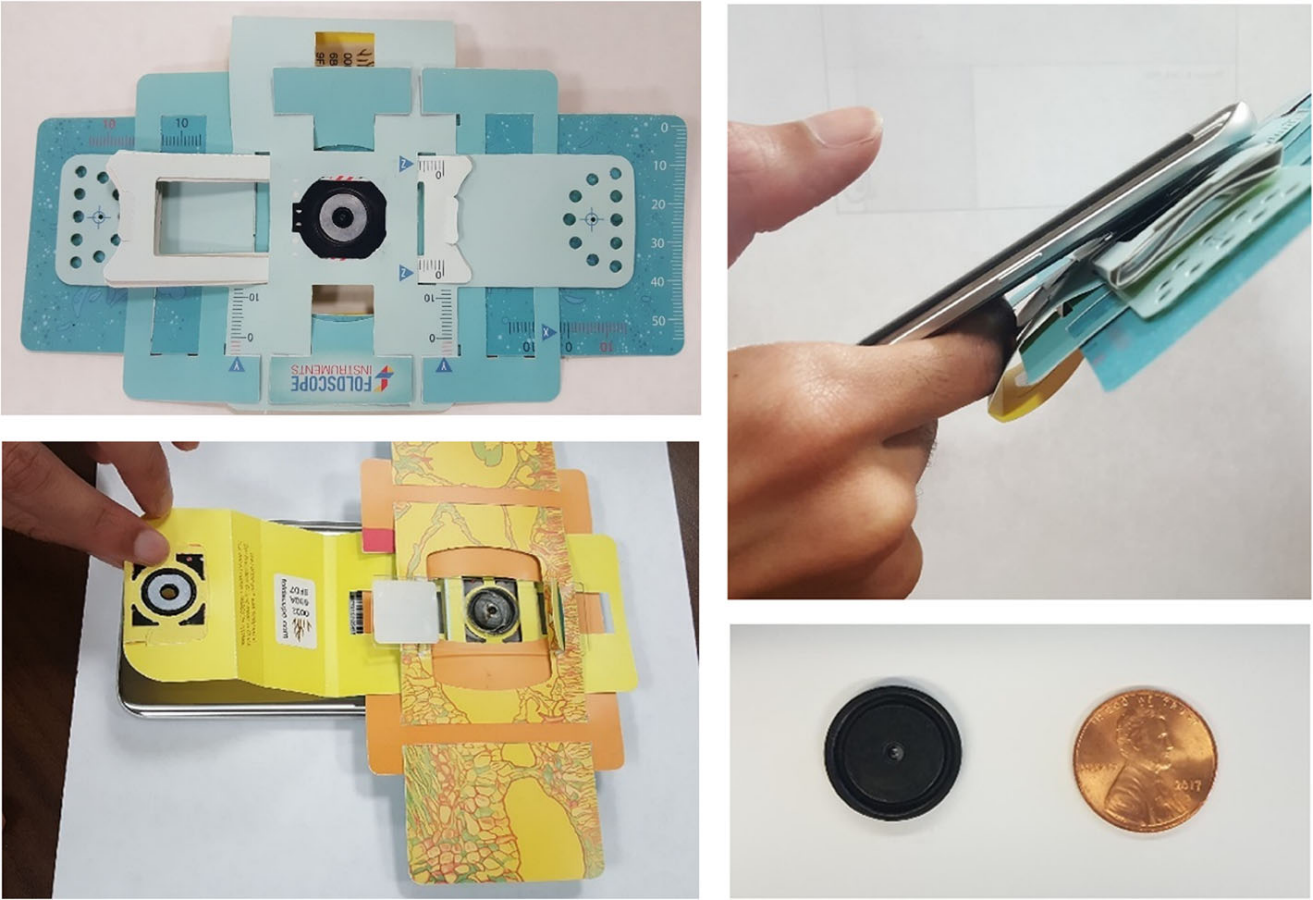
The Foldscope. Assembled paper-based Foldscope (top left). Foldscope phone coupler taped onto a smart phone (top right). Placing the slide within the slide holding apparatus (lower left). Foldscope ball lens comparison with a penny, ball lens is a 2.2 mm borosilicate lens (bottom right)

The slides were rescreened by pathologists (NM or ED) and regions of interest (ROI) were marked for image acquisition. The pathologists were blinded to the original diagnosis and the ROIs chosen were selected to support the diagnosis the current pathologist was considering. Each pathologist identified ROIs on 20 of the 40 slides and acquired photomicrographs. Following conventional microscope imaging, using an Olympus BX-41 or a Leica DM100 microscope at a 10x magnification coupled with a digital camera, the same ROI of the cytology slide was imaged using the Foldscope attached to a Samsung S7 Phone with a 12 MP camera, by one of the authors. This ensured that the same ROI was imaged by both of the methods to be compared. The Foldscope is a $1 origami-paper ball lens phone attachment microscope [3] (Fig. 1). Cervical cytology slides used in this study were inserted into the slide holding plastic-paper apparatus. Imaging was conducted outdoors which provided better illumination than indoor lighting.

After two sets of images (group 1 and group 2) were acquired, 20 images for each set were presented in a randomized sequence to the pathologist who was not involved in image acquisition of the respective slide set (i.e., image group 1 would be reviewed by pathologist 2, and vice versa) (Suppl 1). Pathologists were asked to classify the images in the order presented into one of three categories: Normal, LSIL, and HSIL-Malignant category. HSIL and Malignant categories were combined into a single category since the management approach for both categories is colposcopy and biopsy, and also because increasing the number of images in a single category would increase the robustness of the statistical analysis. Pathologists were given access to Foldscope vs. conventional microscope images 1 week apart, to reduce the chance of observer bias by their previous classification of the same ROI.

### Data analysis

Data collected were used to quantify the agreement between conventional microscopy vs. Foldscope imaging. Two measures of agreement were calculated: percent agreement and the weighted kappa. The percent agreement was calculated by summing the number of concordant observations and diving this sum by the total sample size and multiplying by 100. Percent agreement does not account for agreement that may occur by chance alone [12]. In contrast, kappa statistics correct for chance agreement [12]. A weighted kappa was calculated and reported along with a 95% confidence interval. The null hypothesis that the weighted kappa in the population was zero was evaluated using an exact test. A two-sided *p*-value was reported from the exact test. The result was considered statistically significant if the *p*-value was less than or equal to 0.05. The kappa statistic was interpreted using the classification scheme proposed by Landis and Koch [12]. Briefly, a kappa statistic between 0.8 and 1 translates into an “almost perfect” agreement, while a kappa between 0.6 and 0.8 indicates a “Substantial” agreement. Data were analyzed using SAS 9.4 software (SAS Institute, Inc., Cary, North Carolina).

### Sensitivity and specificity calculation

A 3 X 3 confusion matrix was created with the classes: Normal, LSIL, and HSIL/malignant. The initial classification of each slide made from the clinical pathology laboratory at the time of diagnosis was considered the gold standard, and the results of the assessment made by the pathologists in our study were used to evaluate the performance of the Foldscope by calculating the accuracy, sensitivity, and specificity using the methods described by Tharwat [13].

## Results

**Fig. 2:**
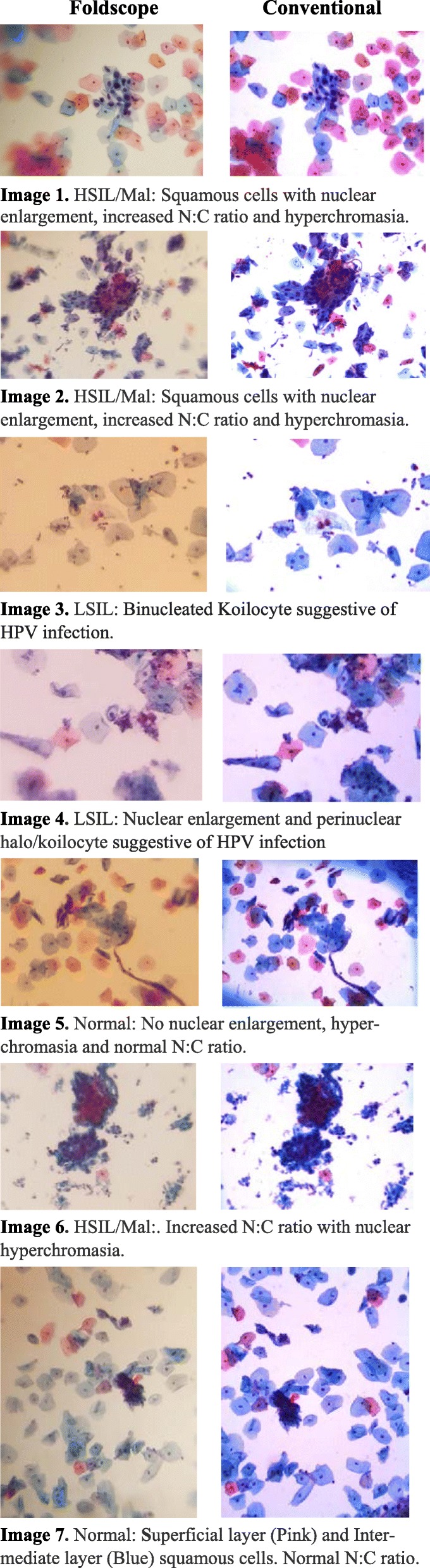
Region of Interest (ROI) images of cervical cytology slides obtained using a Foldscope (left) and a conventional microscope (right)

Comparative images of the same ROIs by the two imaging methodologies are shown in Fig. 2. Presence or lack of increased nucleus/cytoplasm (N:C) ratio, nuclear pleomorphism, and hyperchromasia, along with koilocytic changes were clear and apparent to an extent which allowed for classification of the samples. Clarity variation seemed to be related to the cell density in a cell cluster within a ROI. When multiple cells were present within a single field, an optimal focal plane could sometimes not be obtained using the Foldscope (e.g. Figure 2, Image 6). Diagnostic koilocytic features such as binucleation, as well as perinuclear halos had better resolution and allowed for recognition of LSIL classification (e.g., Fig. 2, image 3 and image 4). We evaluated the agreement of Pap smear classification between the Foldscope vs. conventional microscope imaging. For 16 cases both methods classified the result as HSIL/malignant (Table 1). In one case the Foldscope image was classified as HSIL/malignant while corresponding conventional microscopy was classified as normal. The percent agreement was [(16 + 7 + 7)/40] × 100 = 75%. We observed a weighted kappa of 0.68 (95% confidence interval: 0.49–0.86, exact *p* < 0.0001) for the concordance between the Foldscope and conventional microscopy after correcting for chance agreement (Table 1, and Suppl. Figure 2). This kappa value indicates substantial agreement based on Landis and Koch’s classification [12].

**Table 1:** Agreement between the Foldscope and Conventional Microscopy

Foldscope	Conventional			
	HSIL/MAL	LSIL	Normal	Total
HSIL/MAL	16	2	1	19
LSIL	4	7	2	13
Normal	0	1	7	8
Total	20	10	10	40

Agreement table based on 40 samples. Weighted kappa = 0.68, exact *p* < 0.0001

To calculate the sensitivity and specificity of the Foldscope in the diagnosis of different cervical cytological categories, a 3 × 3 confusion matrix was built, cross-tabulating the initial diagnosis from vs. the classification obtained using the Foldscope (Table 2). The accuracy of the Foldscope was 80%. Sensitivity and specificity were 85 and 90% for the HSIL/Mal category, 80 and 83.3% for LSIL, and 70 and 96.7% for Normal (Table 3).

**Table 2:** Classification performance of the Foldscope compared to the pathology results

Foldscope	Pathological category		
	HSIL	LSIL	Negative
HSIL	17	1	1
LSIL	3	8	2
Negative	0	1	7

*HSIL* high-grade squamous intraepithelial lesion/malignant/positive, *LSIL* low-grade squamous intraepithelial lesion

**Table 3:** Classification performance of the Foldscope compared to the pathology results

Class	Sensitivity (%)	Specificity (%)
HSIL	85	90
LSIL	80	83.3
Negative	70	96.7

*HSIL* high-grade squamous intraepithelial lesion/malignant/positive, *LSIL* low-grade squamous intraepithelial lesion

## Discussion

Most individuals at risk for cervical cancer reside in the developing world [4], which in the majority of cases, lack the resources, infrastructure and/or access to trained pathologists for effective cervical cancer screening. An affordable and easy-to-use diagnostic device could have a significant impact on cervical cancer screening in this setting, especially in remote areas of developing countries [5, 6].

The Foldscope has shown its ability to image parasites such as *Schistosoma haematobium* [14], as well as cervical squamous epithelial cells [10]. This latter study reported a 100% correlation between cytologic characteristics found by the Foldscope and conventional optical microscopy, with a global Cohen kappa index of 0.7, and values of 0.8 for chromatic value, 0.8 for nuclear membrane continuity, and 0.7 for cytoplasmic morphology. The referred study, however, utilized an image station, projecting the image from a Foldscope with an additional condensation system into a dark room [10] thus requiring additional technology and resources that limit its potential application in remote and/or rural settings.

Overall, the Foldscope images were comparable to those obtained with conventional microscope and digital camera setup. For most of the cytology slides (75%), an exact match was observed. In these exact matches, cytologic changes were more clear and classic, compared to discordant cases. The mismatches observed between Normal and LSIL could be attributed to problems with focusing (due to dense cell clusters), limited ROI, as well as subjectivity. Image clarity was the likely reason for the single HSIL/Malignant to Normal discordance observed. Normal cells clumped together with reduced clarity and inspection of a single ROI may have also led to the misclassification.

The limitations of a ROI and subjectivity with the cytologic features may account for the discordances we observed in our study. Most of the mismatches occurred with HSIL/Mal (6 out of 10), and LSIL (5 out of 8). This group of mismatches is the most concerning because of the significant difference in treatment regimens for these two categories. Nevertheless, the sensitivity and specificity for LSIL and HSIL were over 80%. Generally, pathologists screen the entire slide, along with examination of multiple ROIs prior to rendering a diagnosis, which helps in accuracy of classification. The differentiation between LSIL and HSIL may have been difficult with the Foldscope due to the limitation of a single or a few ROIs, and lack of whole slide screening.

In addition, the transition from LSIL to HSIL can itself be subjective in certain groups of cells. The visual evaluation of N:C ratio is inherently subjective and not a quantitative value. Subjectivity and poor reproducibility are not uncommon within pathological classification schemes [15]. In certain classification schemes some categories might not have significant change in the initial diagnostic work-up. This is the case when differentiating between HSIL and malignant/cancer, in which for both categories, the initial diagnostic plan calls for an immediate colposcopy and biopsy. For these reasons and the limited ROI in our project we elected to combine the HSIL and Malignant cytology categories into a single HSIL/Malignant category.

The small visual field, limitations in focusing, along with the inability to make fine movements of the slide under the Foldscope, were the main drawbacks of the Foldscope. The current paper stage does not provide sufficient focusing or slide scanning opportunities, making it a very strenuous task to completely scan/screen a cytology slide. Focusing problems were apparent in the Foldscope as evidenced by large areas with variation in sharpness and clarity even within a single cluster of cells. Lack of sharpness around the focal point and focus constrictions have been previously reported as restrictions of the device [10].

The ball lens microscope is a powerful innovation which may potentially allow for affordable diagnosis of not only cervical cytology but other cytopathological conditions especially with the use of specific Foldscope designs [16]. The lens of the Foldscope is powerful and its ability for classification can be further exploited through computerized analysis. Phone applications with morphometry may allow for rapid classification of cytology and reduce subjective classifications. The development of a staging system as well as improvement in the current focusing system may reduce the scanning obstacles.

## Conclusions

This study demonstrates that the Foldscope lens has substantial agreement with conventional microscope camera. The accuracy of the Foldscope was 80%, with a sensitivity and specificity of 85 and 90% for the HSIL/Mal category, 80 and 83.3%, for LSIL, and 70 and 96.7% for Normal.

This study highlights the usefulness of the Foldscope in cervical cytology.

## Supplementary information

**Additional file 1: Figure S1.** Study Design and flow

**Additional file 2: Figure S2.** Agreement of Foldscope vs. Conventional microscopy

**Additional file 3.** Raw Data

## Data Availability

Raw data is available in Supplementary file 3.

## Abbreviations

DNA: Deoxyribonucleic acid
HPV: Human papillomavirus
HSIL: High-grade squamous intraepithelial lesion
IRB: Institutional review Board
LSIL: Low-grade squamous intraepithelial lesion
N:C: Nucleus/Cytoplasm
ROI: Region of interest
