# Supplementary material for: Evaluating the performance of a low-cost mobile phone attachable microscope in cervical cytology
Source: BMC Womens Health. 2020 Mar 25;20:60. doi: 10.1186/s12905-020-00902-0 (PMC7093980; doi:10.1186/s12905-020-00902-0)

**Suppl 1. Study Design and flow**

Pathologist 2 Image #20- #40 with Conventional Microscope

Pathologist 1 Image #1- #20 with Conventional Microscope


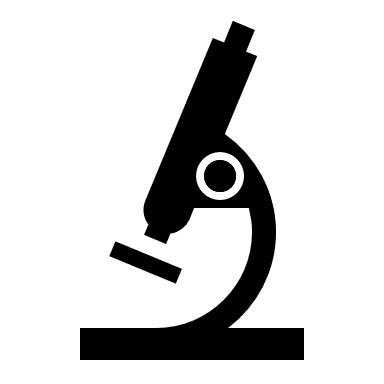


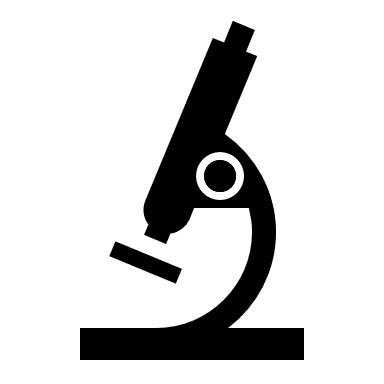


Student Image #1- #20 with Foldscope-Phone

Student Image #20- #40 with Foldscope-Phone


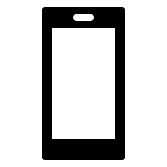

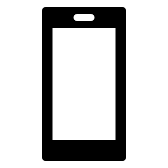


Pathologist 2 Classify #1- #20 Foldscope Image Classification

Pathologist 1 Classify #20- #40 Foldscope Image

Agreement Analysis

Suppl 2


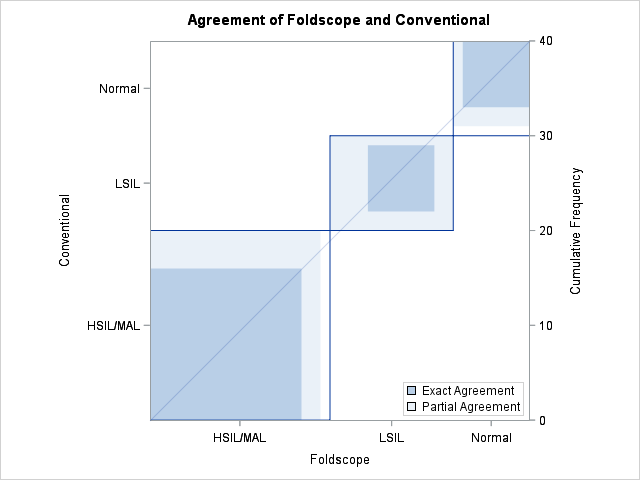

Supplement: Supplementary file 1 — Additional file 1: Figure S1. Study Design and flow [file 12905_2020_902_MOESM1_ESM.docx]
