# Supplementary figures and images for: Evaluating the performance of a low-cost mobile phone attachable microscope in cervical cytology
Source: BMC Womens Health. 2020 Mar 25;20:60. doi: 10.1186/s12905-020-00902-0 (PMC7093980; doi:10.1186/s12905-020-00902-0)

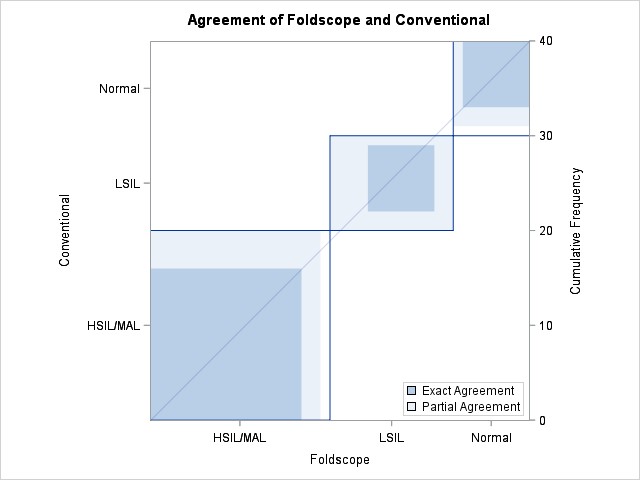

Supplement: Supplementary file 2 — Additional file 2: Figure S2. Agreement of Foldscope vs. Conventional microscopy [file 12905_2020_902_MOESM2_ESM.jpg]
