# Supplementary material for: Evaluating the performance of a low-cost mobile phone attachable microscope in cervical cytology
Source: BMC Womens Health. 2020 Mar 25;20:60. doi: 10.1186/s12905-020-00902-0 (PMC7093980; doi:10.1186/s12905-020-00902-0)
Supplement: Supplementary file 3 — Additional file 3. Raw Data [file 12905_2020_902_MOESM3_ESM.docx]

Suppl 3

Raw Data

| Foldscope 1 | Actual |  |  | Foldscope G2 | **Actual** |  |
| --- | --- | --- | --- | --- | --- | --- |
| N | Neg |  |  | HSIL/Mal | HGSIL |  |
| HSIL/MAL | Neg | -1 |  | LSIL | LGSIL |  |
| N | Neg |  |  | HSIL/Mal | HGSIL |  |
| HSIL/MAL | Pos |  |  | N | Neg |  |
| HSIL/MAL | Pos |  |  | N | Neg |  |
| HSIL/MAL | Pos |  |  | LSIL | LGSIL |  |
| HSIL/MAL | Pos |  |  | HSIL/Mal | HGSIL |  |
| HSIL/MAL | Pos |  |  | HSIL/Mal | LGSIL | -1 |
| LSIL | Neg | -1 |  | LSIL | HGSIL | -1 |
| HSIL/MAL | HGSIL |  |  | N | Neg |  |
| LSIL | LGSIL |  |  | N | LGSIL | -1 |
| HSIL/MAL | HGSIL |  |  | LSIL | LGSIL |  |
| LSIL | LGSIL |  |  | HSIL/Mal | Pos |  |
| HSIL/MAL | Pos |  |  | LSIL | HGSIL | -1 |
| LSIL | Pos | -1 |  | HSIL/Mal | HGSIL |  |
| HSIL/MAL | HGSIL |  |  | N | Neg |  |
| LSIL | LGSIL |  |  | HSIL/Mal | Pos |  |
| LSIL | Neg | -1 |  | HSIL/Mal | HGSIL |  |
| N | Neg |  |  | LSIL | LGSIL |  |
| LSIL | LGSIL |  |  | HSIL/Mal | Pos |  |
|  |  |  |  |  |  |  |
|  | mismatch | -4 |  |  | Mismatch | -4 |

Group1

|  | Conventional Pathol1 | Foldscope Pathol2 | Conventional Pathol2 |
| --- | --- | --- | --- |
| 1 | N | N | N |
| 2 | N | HSIL/MAL | LSIL |
| 3 | N | N | N |
| 4 | HSIL/MAL | HSIL/MAL | HSIL/MAL |
| 5 | HSIL/MAL | HSIL/MAL | HSIL/MAL |
| 6 | HSIL/MAL | HSIL/MAL | HSIL/MAL |
| 7 | HSIL/MAL | HSIL/MAL | HSIL/MAL |
| 8 | HSIL/MAL | HSIL/MAL | HSIL/MAL |
| 9 | N | LSIL | N |
| 10 | HSIL/MAL | HSIL/MAL | HSIL/MAL |
| 11 | LSIL | LSIL | LSIL |
| 12 | LSIL | HSIL/MAL | LSIL |
| 13 | LSIL | LSIL | LSIL |
| 14 | HSIL/MAL | HSIL/MAL | HSIL/MAL |
| 15 | HSIL/MAL | LSIL | LSIL |
| 16 | HSIL/MAL | HSIL/MAL | HSIL/MAL |
| 17 | LSIL | LSIL | LSIL |
| 18 | N | LSIL | N |
| 19 | N | N | N |
| 20 | LSIL | LSIL | LSIL |

| Inter Pathologist Correlation |  | Pathol2 Self Foldoscope Vs. Conventional |
| --- | --- | --- |
|  |  |  |
| Perfect Correlation : 15/20 |  | Correlation: 16/20 |
| No Correlation: 5/20 |  | No Correlation: 4/20 |

| GROUP 2 | Convential Pathol | Foldscope Pathol1 | Conventional Pathol1 |
| --- | --- | --- | --- |
| 1 | HSIL/MAL | HSIL/Mal | HSIL/Mal |
| 2 | LSIL | LSIL | LSIL |
| 3 | HSIL/MAL | HSIL/Mal | HSIL/Mal |
| 4 | N | N | N |
| 5 | N | N | N |
| 6 | LSIL | LSIL | LSIL |
| 7 | HSIL/MAL | HSIL/Mal | HSIL/Mal |
| 8 | LSIL | HSIL/Mal | HSIL/Mal |
| 9 | HSIL/MAL | LSIL | LSIL |
| 10 | N | N | N |
| 11 | LSIL | N | LSIL |
| 12 | LSIL | LSIL | LSIL |
| 13 | HSIL/MAL | HSIL/Mal | HSIL/Mal |
| 14 | HSIL/MAL | LSIL | HSIL/Mal |
| 15 | HSIL/MAL | HSIL/Mal | HSIL/Mal |
| 16 | N | N | N |
| 17 | HSIL/MAL | HSIL/Mal | LSIL |
| 18 | HSIL/MAL | HSIL/Mal | HSIL/Mal |
| 19 | HSIL/MAL | LSIL | HSIL/Mal |
| 20 | HSIL/MAL | HSIL/Mal | HSIL/Mal |

| Inter Pathologist Correlation |  | Pathol2 Self Foldoscope Vs. Conventional |
| --- | --- | --- |
|  |  |  |
| Perfect Correlation : 15/20 |  | Correlation: 16/20 |
| No Correlation: 5/20 |  | No Correlation: 4/20 |
